# Vertical disc tilt and features of the optic nerve head anatomy are related to visual field defect in myopic eyes

**DOI:** 10.1038/s41598-019-38960-6

**Published:** 2019-03-05

**Authors:** Hae-Young Lopilly Park, Yong Chan Kim, Younhea Jung, Chan Kee Park

**Affiliations:** 1 Department of Ophthalmology and Visual Science, Seoul St. Mary’s Hospital, College of Medicine, The Catholic University of Korea, Seoul, South Korea; 2 Department of Ophthalmology and Visual Science, St. Mary’s Hospital, College of Medicine, The Catholic University of Korea, Seoul, South Korea

## Abstract

Myopia is significantly growing eye disease which accompanies various ocular pathologies including glaucoma. Understanding visual field (VF) and retinal nerve fiber layer (RNFL) damage observed in myopic eyes is important. Therefore, we evaluated optic disc margin anatomy using spectral-domain (SD) optical coherence tomography (OCT). We enrolled 40 healthy myopic patients and 64 myopic patients with RNFL defects in the superonasal region of the optic disc on red-free photographs and OCT. Optic disc stereophotographs were co-localized to SD-OCT images, and we analyzed the frequency with which the disc margin identified in photographs coincided with Bruch’s membrane (BM) opening, BM/border tissue, or border tissue. For each scan, the presence of border tissue overhang beyond the clinical disc margin and the end of Bruch’s membrane was identified. Among 64 myopic eyes with RNFL defects, 42 had corresponding inferotemporal VF defects. Border tissue overhang was found from 25 to 60% of myopic eyes with RNFL defect at all clock positions. However, border tissue overhang was found from 86 to 97% at 1, 2, 11 and 12 o’clock position in myopic eyes with VF defects. This was significantly different from myopic healthy eyes. We suggest that the OCT structure at the disc margin might contribute to VF damage in myopic patients.

## Introduction

The frequency of myopia and high myopia is rapidly increasing among young populations, especially in East Asians^1–3^. Various sight-threatening ocular diseases accompany myopia. For example, myopia, particularly high myopia, is a well-known risk factor for glaucoma^4–7^. Myopic glaucoma patients were significantly younger than non-myopic glaucoma patients in our previous study^8^, and thus myopia is rapidly becoming a major public health problem in this young population. However, accurate diagnosis of glaucoma in myopic eyes is challenging in clinical practice because the optic disc anatomy is different from eyes without myopia. Disc tilting and large amounts of peripapillary atrophy (PPA), and larger disc area, for example, can disturb the interpretation of standard glaucoma tests^9^. There are reports that myopic degeneration can also cause glaucomatous-like visual field (VF) defects, which can minimally or not progress as glaucoma^10,11^. Therefore, myopic patients need to be followed over time to confirm true glaucoma^12^. Even in true glaucoma patients with myopia, recent studies have reported that glaucoma with myopia progresses more slowly than glaucoma without myopia^10,13–15^. Thus, confirmation of a glaucoma diagnosis and detecting progression can be very difficult in myopic eyes, even after longitudinal follow-up. Recently, application of spectral-domain (SD) optical coherence tomography (OCT) provides adjunctive tool for identifying glaucoma in myopic eyes^16,17^.

The primary site of damage in glaucoma is thought to be the optic nerve head (ONH). Retinal ganglion cell axons pass through the lamina cribrosa and pass ONH structures, such as the sclera lip, border tissue of Elschnig, and sometimes Bruch’s membrane (BM), as they bend horizontally to the retina. Investigations have sought out the association between glaucomatous damage and these ONH structures^18–20^. Glaucomatous eyes with focal damage frequently have border tissue that extends into the ONH at the inferotemporal disc, where most glaucomatous damage occurs^19^. These microstructures in the region of peripapillary atrophy have been attributed to glaucomatous damage or the progression of glaucoma^21,22^ and also to myopia^23^. We decided to look at ONH structures using SD-OCT in myopic eyes to evaluate the association between the ONH structures and the VF/RNFL damage observed in myopic eyes, which is not a classic form of glaucoma damage. In this way, we hope to suggest the clinical relevance of myopic VF defects or RNFL changes and their difference from glaucomatous damage. Additionally, better understanding of the relationship of ONH anatomy with the VF in myopic eyes may enable clinicians to better identify eyes with glaucoma.

## Results

We analyzed a total of 134 eyes with myopia that met the inclusion and exclusion criteria. Of those, we further investigated 104 (77.6%) eyes excluding the others due to difficulties or disagreements on the disc margin delineation. We included healthy myopic eyes (n = 40), myopic eyes with superonasal (SN) RNFL thinning, but no VF defects (n = 22), and myopic eyes with SN RNFL thinning with corresponding VF defects (n = 42) in the analysis. Baseline characteristics, except for mean deviation and pattern standard deviation of the VF, were similar between the groups, as shown in Table 1. The mean deviation and pattern standard deviation of the VF were significantly different between myopic eyes with and without VF defects. The morphological features of the optic disc were significantly different between the myopic eyes with and without VF defects in terms of vertical disc tilt degree measured from the HRT images (*P* = 0.036). The RNFL thickness showed significant differences among the three groups in the nasal quadrant at clock hours 1, 2, 11, and 12, as shown in Table 2. Myopic eyes with and without VF defects had significantly different RNFL thickness in the superior and nasal quadrants at clock hours 1, 2, 11, and 12.

**Table 1:** Demographic of enrolled patients in this study.

	Myopic control eyes (n = 40)	Myopic eyes without VF defects (n = 22)	Myopic eyes with VF defects (n = 42)	*P* value^a^	*P* value^b^
Age, years	42.54 ± 13.74	39.04 ± 13.41	36.42 ± 13.51	0.109^*^	0.863^*^
Gender, Male:Female	19:21	04:18	14:28	0.102^†^	0.093^†^
Central corneal thickness, µm	538.36 ± 37.54	540.80 ± 40.36	537.19 ± 26.09	0.155^*^	0.420^*^
Spherical equivalent, diopters	−5.35 ± 2.24	−6.17 ± 2.91	−5.51 ± 2.18	0.148^*^	0.969^*^
Axial length, mm	25.93 ± 1.32	26.14 ± 1.37	25.75 ± 1.31	0.720^*^	0.654^*^
Untreated intraocular pressure, mmHg	15.29 ± 2.59	15.45 ± 2.63	15.24 ± 2.74	0.623^*^	0.720^*^
Mean deviation of VF, dB	−1.94 ± 2.30	−2.33 ± 2.10	−5.58 ± 5.08	<0.001^*^	0.048^*^
Pattern standard deviation of VF, dB	3.27 ± 1.32	3.65 ± 2.67	7.49 ± 4.23	<0.001^*^	0.008^*^
Optic disc morphological features					
Ovality ratio	1.38 ± 0.23	1.40 ± 0.29	1.33 ± 0.28	0.070^*^	0.310^*^
Direction of disc tilt, inferotemporal, n (%)	28 (70.0%)	13 (59.1%)	28 (66.7%)	0.684^†^	0.591^†^
Disc torsion degree	16.54 ± 9.54	18.97 ± 10.08	15.73 ± 7.96	0.383^*^	0.411^*^
Disc-fovea angle	8.12 ± 3.65	7.52 ± 3.66	8.43 ± 3.11	0.520^*^	0.470^*^
Vertical tilt measured from HRT images	0.02 ± 0.01	0.02 ± 0.02	0.06 ± 0.05	0.036^*^	0.047^*^
Horizontal tilt measured from HRT images	0.04 ± 0.03	0.04 ± 0.04	0.02 ± 0.02	0.112^*^	0.268^*^

VF = visual field.

^*^One-way ANOVA with multiple comparison.

^†^Chi-square test.

^a^Comparison between three groups.

^b^Comparison between myopic eyes with and without VF defects.

**Table 2:** The retinal nerve fiber layer thickness measured using peripapillary optical coherence tomograph.

	Myopic control eyes (n = 40)	Myopic eyes without VF defects (n = 22)	Myopic eyes with VF defects (n = 42)	*P* value^a^	*P* value^b^
Average	90.00 ± 17.24	85.85 ± 11.20	74.05 ± 12.68	<0.001	0.014
Quadrant					
Superior	93.84 ± 17.46	89.70 ± 23.92	65.15 ± 16.95	0.122	0.001
Nasal	80.24 ± 16.34	77.95 ± 8.55	50.90 ± 13.03	0.380	0.050
Inferior	101.15 ± 18.86	106.20 ± 20.96	99.35 ± 19.93	0.424	0.296
Temporal	87.54 ± 19.74	91.60 ± 22.56	80.75 ± 24.89	0.567	0.911
Clock hour					
12 Superior	78.00 ± 20.87	79.84 ± 30.47	55.89 ± 17.47	<0.001	0.005
11	107.82 ± 24.42	113.79 ± 30.85	85.31 ± 26.55	<0.001	0.004
10	89.13 ± 21.83	91.00 ± 26.83	86.94 ± 36.13	0.236	0.697
9 Temporal	67.95 ± 13.52	75.84 ± 18.23	67.68 ± 21.65	0.174	0.778
8	94.00 ± 18.41	93.21 ± 26.74	89.78 ± 27.68	0.380	0.701
7	130.73 ± 27.00	140.05 ± 34.78	130.00 ± 29.18	0.573	0.341
6 Inferior	103.63 ± 19.45	104.37 ± 28.15	97.10 ± 31.15	0.248	0.456
5	80.04 ± 13.08	74.73 ± 18.07	69.26 ± 19.52	0.134	0.376
4	69.18 ± 8.22	57.57 ± 8.52	53.68 ± 9.47	0.202	0.191
3 Nasal	66.45 ± 12.88	64.52 ± 9.14	50.52 ± 11.52	0.326	0.244
2	77.68 ± 12.35	71.42 ± 12.22	53.89 ± 11.44	<0.001	0.028
1	88.50 ± 15.40	89.57 ± 18.55	58.21 ± 19.48	<0.001	<0.001

VF = visual field.

^*^One-way ANOVA with multiple comparison.

^†^Chi-square test.

^a^Comparison between three groups.

^b^Comparison between myopic eyes with and without VF defects.

**Figure 1:**
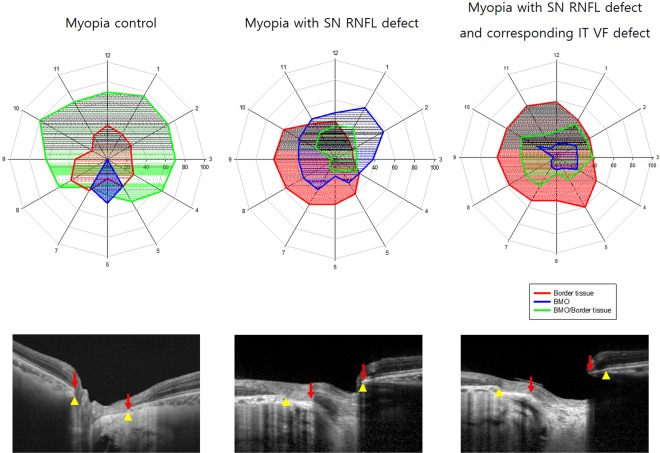
Polar plots with connected points showing the frequency of spectral domain optical coherence tomography (SD-OCT) structures corresponding to the optic disc margin by clock hour for myopic healthy subjects, myopic subjects with retinal nerve fiber layer defect (RNFL) only, and myopic subjects with both RNFL defect and corresponding visual field defects. The distance from the origin at each clock hour represents the frequency of each SD-OCT structure analyzed. The optic disc margin structure in myopic subjects with both RNFL/VF defects showed different anatomical profiles compared to myopic subjects without VF defects at the 11, 12, 1, and 2 o’clock positions. SN, superonasal; IT, inferotemporal; RNFL, retinal nerve fiber; VF, visual field. Yellow arrowhead indicates the end of Bruch’s membrane amd red arrow indicates the end of border tissue.

The Jaccard indices for delineating the clinical disc margin was 0.94 showing good agreement between observers. The SD-OCT structure most commonly coinciding with the clinical disc margin in healthy myopic controls was the BM with border tissue together (Fig. 1). It was followed by border tissue, and BM alone. However, in myopic eyes with SN RNFL defect, the SD-OCT structure most commonly observed to coincide with the clinical disc margin was the border tissue at the inferotemporal (IT) region of the ONH and BMO at the SN region of the ONH. There were significant differences between the healthy myopic controls and myopic eyes with SN RNFL defect in the frequency of SD-OCT structures identified as the disc margin (*P* < 0.05 at all clock hours). Myopic eyes with SN RNFL/IT VF defects showed statistically less BMO and frequent border tissue on the clinical disc margin at the SN region than myopic eyes without VF defects (*P* < 0.05 in 1, 2, 11, and 12 o’clock positions).

**Figure 2:**
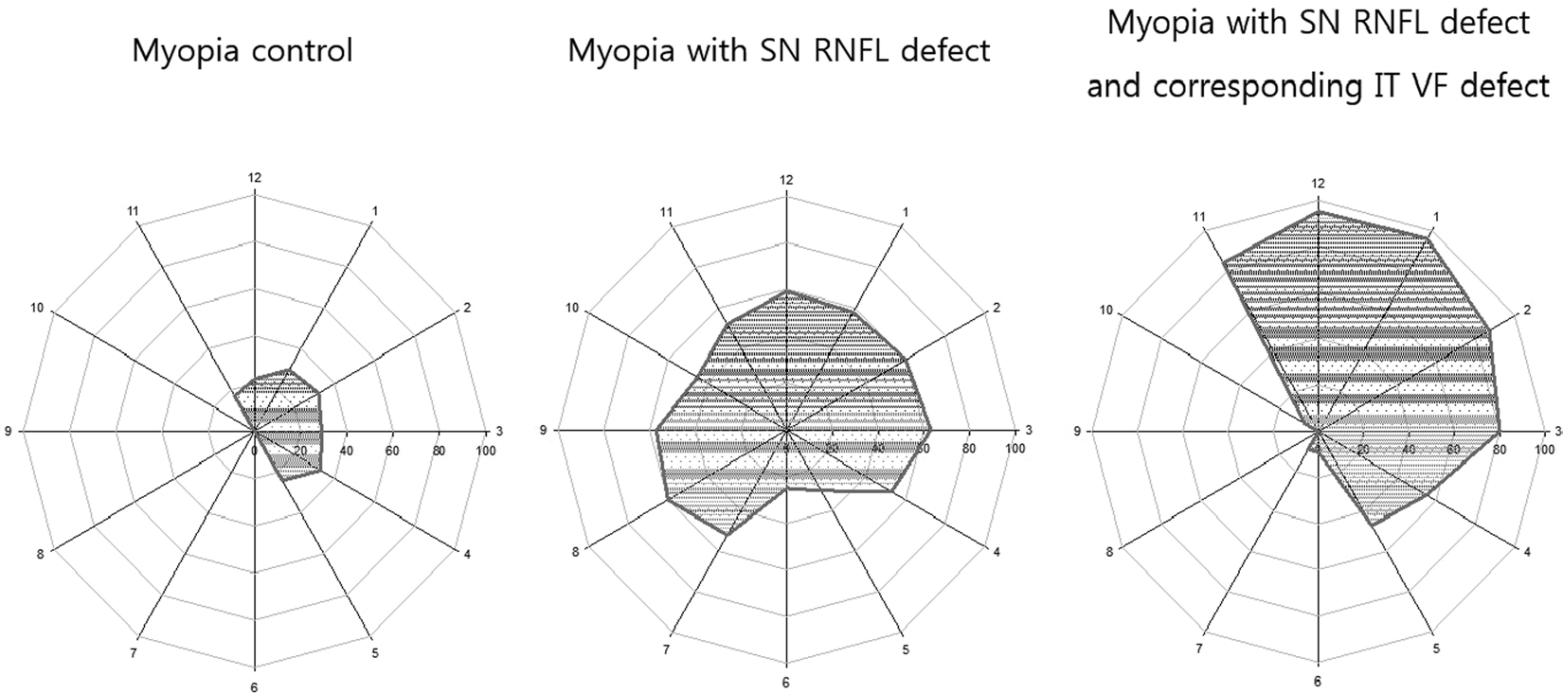
Polar plots with connected points showing the frequency of border tissue overhang by clock hour position for myopic control subjects, myopic subjects with retinal nerve fiber layer defect (RNFL) only, and myopic subjects with RNFL defect and corresponding visual field defects. The distance from the origin at each clock hour position represents the frequency. SN, superonasal; IT, inferotemporal; RNFL, retinal nerve fiber; VF, visual field.

The frequency of border tissue overhang was significantly different between groups (Fig. 2). In the myopic eyes with SN RNFL defect, we found border tissue overhang in 25–60% of eyes at all clock positions. However, this was significantly reduced in healthy myopic eyes to less than 34% around the optic disc (*P* < 0.05 at all clock hours). In myopic eyes with SN RNFL/IT VF defects, border tissue overhang was found in 86–97% of eyes at the 1, 2, 11 and 12 o’clock positions.

## Discussion

Several recent publications showed that the clinical disc margin has various configurations and structures related to ONH anatomy^18,19^. However, the clinical significance of those findings remains under investigation. Reis *et al*. demonstrated that BM overhang is commonly present in human eyes, which is not visible clinically, and the clinical disc margin consists of a combination of the BM edge and some part of the border tissue^19^. Perhaps, the location of glaucomatous axonal damage could be driven by the anatomic characteristics of ONH structures at the optic disc margin. The study by Reis *et al*. showed a tendency for glaucomatous eyes to have more externally oblique border tissue inferotemporally than controls, which could contribute to an oblique course for the retinal ganglion cell axons through the sclera and cause preferential axonal damage in glaucoma. However, their observation did not reach statistical significance to confirm those findings. BM overhang was not statistically different between the normal and glaucoma groups, but was observed in 85–86% of the myopic eyes in both groups^18,23^. In the present study, we found a distinct difference between myopic control eyes and myopic eyes with RNFL or VF damage in terms of the corresponding ONH structure at the clinical disc margin (as observed by OCT) and border tissue overhang. In myopia, the optic disc undergoes morphological changes such as optic disc tilt, torsion, formation of PPA, and sometimes optic canal expansion^10,24,25^. The optic nerve is inserted into the globe in a skewed direction as eyeball elongation occurs^26,27^. Thus, it is unsurprising that the ONH structure observed by OCT is different in myopic eyes than in normal controls. We further found that the ONH anatomy at the clinical disc margin differed between myopic eyes with and without VF defects and RNFL damage. Myopic eyes with IT VF defects had reduced RNFL thickness in the corresponding nasal/SN regions. The ONH structure in myopic eyes with VF defects in the SN and nasal side of the optic disc showed that the BMO corresponded to the clinical disc margin with statistically lower frequency, and there was frequent border tissue overhang. We suggest that this specific optic disc margin anatomy or the presence border tissue overhang without BM could cause preferential axonal damage at that location observed in myopic eyes. Myopic eyes with VF defects enrolled in our study had IT VF defects between the 20 and 60 degree regions in the full field perimetry and we think this could be related to the optic disc margin anatomy and the border tissue overhang at the SN region.

**Figure 3:**
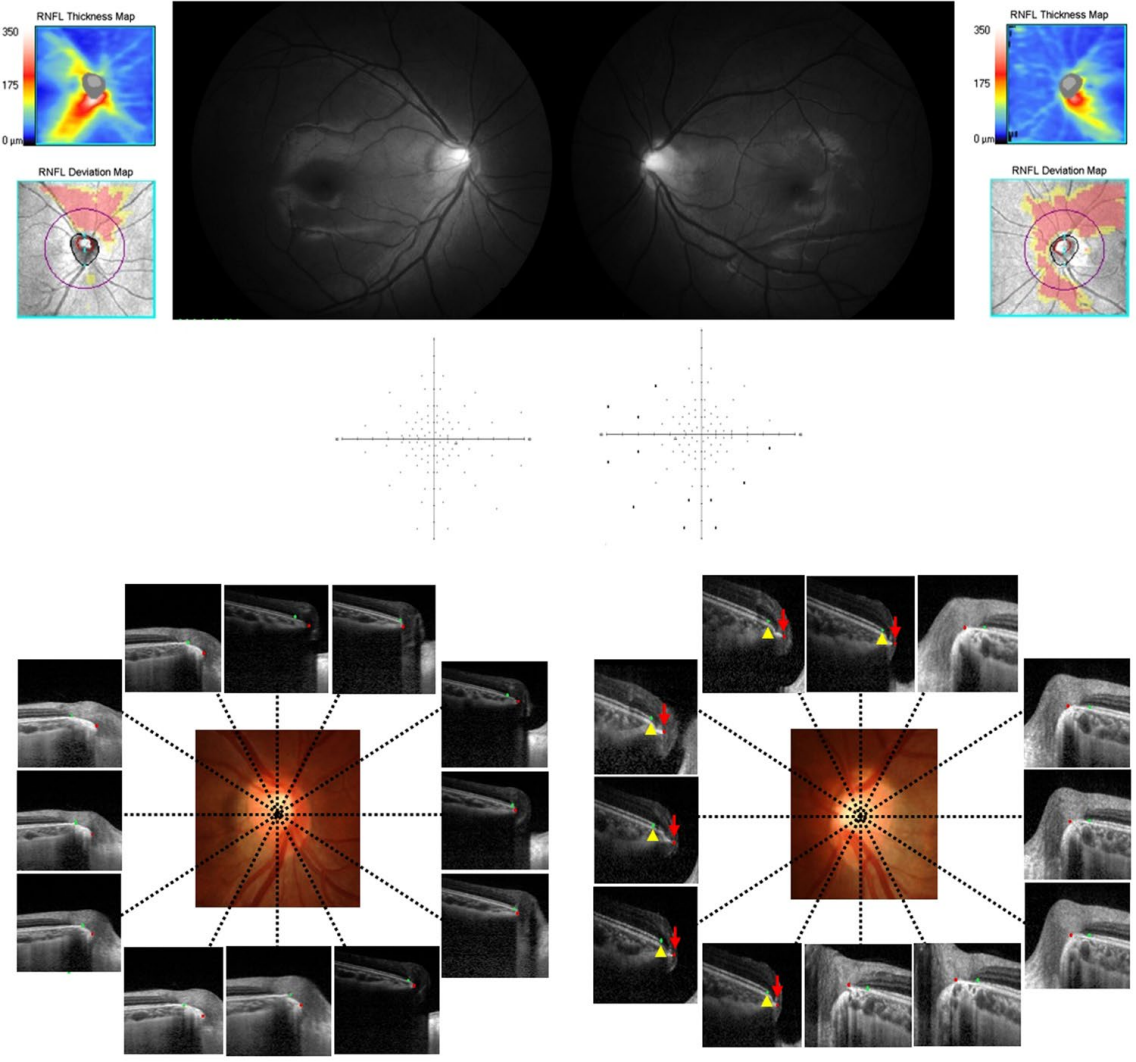
Typical representative case with myopia enrolled in the present study. This patient exhibits superonasal retinal nerve fiber layer (RNFL) damage on red-free photography and optical coherence tomography, which is more prominent in the left eye. Only an inferotemporal VF defect is present in the left eye on the full-field suprathreshold test in the 20–60 degree region. The optic disc anatomy shows border tissue overhang end in the superonasal and nasal region where RNFL damage corresponds to VF defects in the left eye. Green glyph, Bruch’s membrane; Red glyph, border tissue end; Yellow arrowhead and red arrow indicates regions with border tissue overhang beyond the Bruch’s membrane end.

As seen in the representative case in Fig. 3, the eye of a myopic patient had RNFL defects at the SN and nasal areas on the red-free photography and OCT. The IT VF defect is found in only the left eye on the full field suprathreshold test. The OCT scans of the ONH show that border tissue overhang at the 12, 11, 10, 9, 8, and 7 o’clock positions is observed in only the left eye, where it corresponds to the IT and temporal VF defect location. If the RNFL defect and the corresponding VF defect result from axonal damage to the RNFL fibers caused by the ONH structure, this particular group of myopic patients should be classified as having a different category of disease, which we call myopic optic neuropathy. Confirming the further clinical course of patients with myopic optic neuropathy will require longitudinal investigations. However, we need to be cautious in considering VF defects and RNFL damage in myopic eyes since the damage may also occur at the level of the ONH related to myopic changes in this region, similar to glaucomatous damage but in a different location.

Previous studies investigated the ONH structures at the temporal region of the optic disc, where PPA is present in most myopic eyes. The most frequent ONH anatomy found in the PPA region in myopic eyes was PPA without BM, which could result from axial elongation^23,28,29^. We think the border tissue overhang in the SN and nasal region of the optic disc is caused by the disc tilting that occurs in myopic changes, additional to scleral stretching and axial elongation. Usually, an IT direction of disc tilt is present in myopic eyes^8,30,31^. Scleral stretching and the development of PPA in the temporal disc region caused by disc tilt could contribute to regional susceptibility to axonal damage in myopic eyes. However, bending of the ONH structures by the border tissue overhang at the nasal disc region might also contribute to the regional susceptibility we observed in myopic patients with myopic optic neuropathy. A previous study by Akagi, T. *et al*. reported that the scleral bending angle was related to the degree of RNFL damage in myopic eyes. They suggested that direct mechanical damage to the nerve fibers caused by the sharp angle of scleral bending and excessive tension to the fiber could contribute to the damage^32^. However, they also suggested that direct damage is not the sole mechanism for VF defects in myopic eyes. The location of scleral bending and the VF defect did not match completely in some patients. Further investigation is needed to determine other additional contributing factors in VF damage in myopic patients. The amount and degree of the border tissue overhang may also be related to the degree of VF damage, which may be further investigated.

Our study had several limitations. First, we included glaucoma patients from a single ethnic group. Thus, these results might not be applicable to all patients with glaucoma. Second, the study was a cross-sectional study. Longitudinal studies are required to determine the cause and effect relationship between ONH structures and the location of myopic VF and RNFL damage. Finally, we tried to include only myopic patients with a consistent pattern of VF and RNFL defects excluding typical VF defects at the Bjerrum area or findings suggesting glaucoma. We also excluded pathologic myopia patients with other retinal lesions. However, myopic eyes are difficult to assess in certain cases, and despite our careful examination, the potential for misclassification must be considered when interpreting our findings. Additionally, we excluded myopic patients with abnormal disc morphology, only including eyes with enlarged cup-to-disc ratio not exceeding 0.7. This should be considered when applying our findings to myopic patients.

In summary, we found a typical pattern of VF and RNLF damage in myopic patients, which differed from classical glaucomatous change. Such patients have RNFL defects at the SN and nasal regions of the optic disc with corresponding IT VF defects in the 20–60 degree VF region as determined by full-field VF tests. In the corresponding SN and nasal optic disc region, the ONH structures that coincide with the clinical disc margin were distinct from those in myopic patients without VF damage, and border tissue overhang was frequent in this region. We suggest that this ONH anatomy could contribute to the RNFL and VF damage observed in myopic patients.

## Methods

### Subjects

This prospective study began in March 2012. It was approved by the Institutional Review Board of Seoul St. Mary’s Hospital, Seoul, South Korea and followed the tenets of the Declaration of Helsinki. We obtained written informed consent from consecutive patients who met the eligibility criteria and were willing to participate in the study.

We enrolled myopic patients referred to the glaucoma clinic at Seoul St. Mary’s Hospital for glaucoma screening. Healthy myopic controls were enrolled from the dry eye clinic at the same hospital. We defined myopia as a spherical equivalent less than −2.00 diopters and an axial length greater than 24.0 mm. Each participant underwent a comprehensive ophthalmic assessment, including measurement of best-corrected visual acuity, refraction, slit-lamp biomicroscopy, gonioscopy, Goldmann applanation tonometry, central corneal thickness using ultrasound pachymetry (Tomey Corporation, Nagoya, Japan), determination of axial length using ocular biometry (IOL Master; Carl Zeiss Meditec, Dublin, CA, USA), dilated stereoscopic examination of the optic disc and fundus, color disc photography, red-free RNFL photography (Kowa, Tokyo, Japan), confocal scanning laser ophthalmoscopy using the Heidelberg retina tomograph (HRT) III (Heidelberg Engineering, Heidelberg, Germany), and OCT (Cirrus OCT; Carl Zeiss Meditec). For this study, patients performed two VF tests: the Humphrey VF examination (Carl Zeiss Meditec) using the 24–2 Swedish interactive threshold algorithm standard program (Goldmann size III stimulus) and a full-field 81 point screening test using the suprathreshold strategy.

Patients were required to meet the following inclusion criteria: a best-corrected visual acuity of ≥20/40, mean deviation (MD) better than −12.00 decibels (dB) on the Humphrey 24–2 VF test, and consistently reliable VFs (defined as a false-negative rate of <15%, a false positive rate of <15%, and fixation losses of <20%). Patients were excluded when glaucoma was suspected: glaucomatous optic disc changes (such as diffuse or localized rim thinning, a notch in the rim, or a vertical cup-to-disc ratio higher than 0.7) and glaucomatous VF loss (defined as the consistent presence of a cluster of ≥3 non-edge points on the pattern deviation plot with a probability of occurring in <5% of the normal population, with one of the points having the probability of occurring in <1% of the normal population, a pattern standard deviation with *P* < 5%, or a Glaucoma Hemifield Test result outside the normal limits in a consistent pattern on Humphrey 24–2 tests), confirmed by two glaucoma specialists (Y.J. and C.K.P.). Additionally, patients were excluded on the basis of any of the following criteria: an axial length >30 mm; a history of any retinal disease; a history of eye trauma or surgery; incisional surgery or laser procedure; another optic nerve disease besides glaucoma; or a history of systemic or neurological diseases that might affect the VF. Normal controls had a normal eye examination with an intraocular pressure less than 21 mmHg and a normal visual field defined using the Glaucoma Hemifield Test, mean deviation, and pattern standard deviation within normal limits. Only one eye was randomly chosen for analysis.

**Figure 4:**
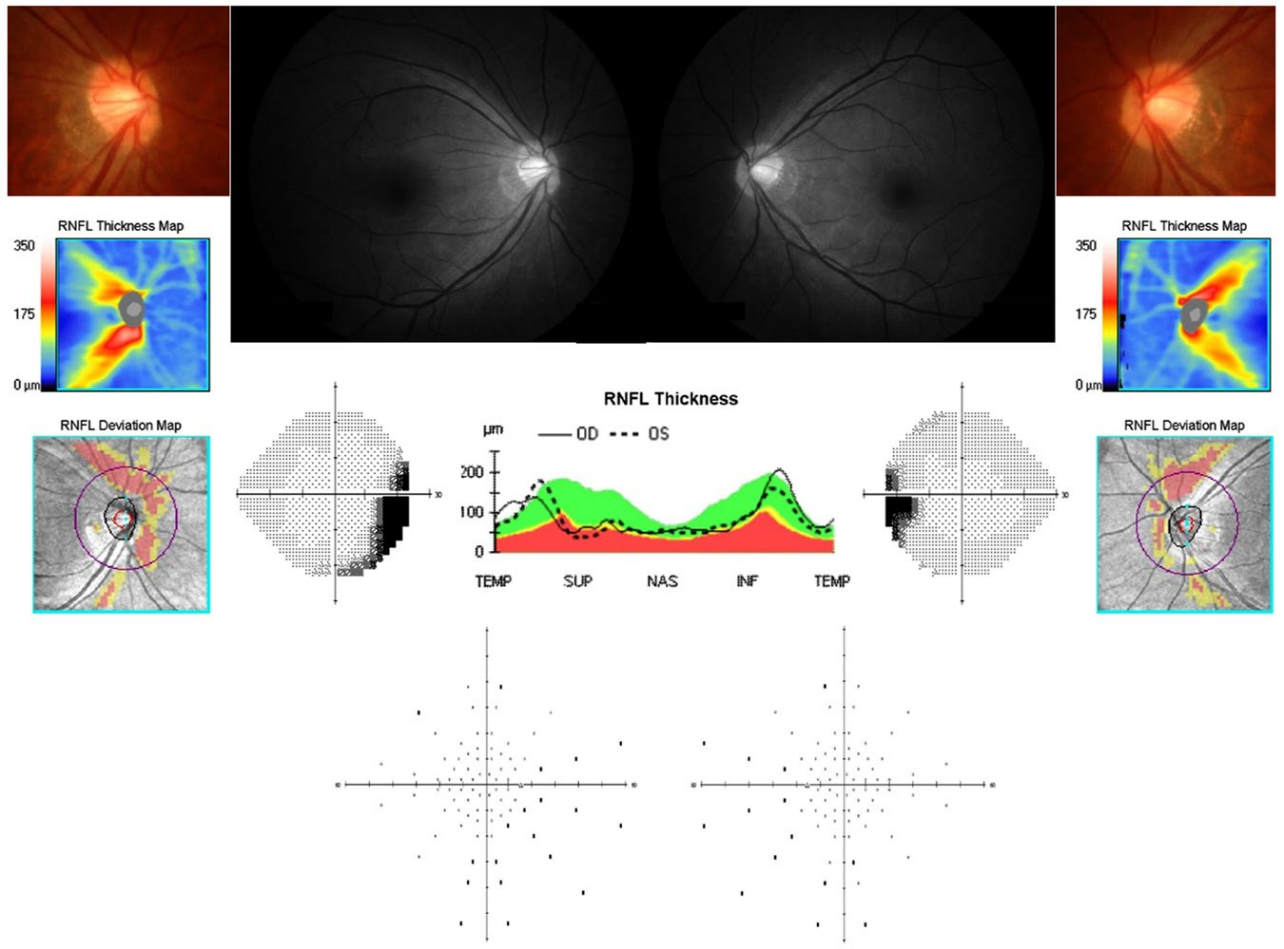
Typical representative myopia case with retinal nerve fiber layer (RNFL) defect enrolled in the present study. Decreased RNFL is visible at the superonasal or nasal region on red-free photography or optical coherence tomography (OCT); however, no focal RNFL defects are present on photography/OCT nor asymmetry of the RNFL between the eyes on the TSNIT map of OCT. Among these patients, when inferotemporal visual field (VF) defect was observed on Humphrey 24–2 tests or full-field 81 point screening tests, they were classified as having both RNFL and VF defects. This representative case shows inferotemporal VF defect in the right eye, whereas only blind spot enlargement in the left eye.

Some of the myopic patients had additional findings of an enlarged cup-to-disc ratio (not exceeding 0.7) or asymmetry of the cup-to-disc ratio between the eyes. However, only patients with no definite evidence of glaucomatous damage after detailed glaucoma evaluation were included in the study. Myopic patients without any abnormalities in the RNFL and VF were classified as myopic healthy control group. Myopic patients with decreased RNFL at the superonasal (SN) region on red-free photography or OCT, which were previously reported to be false-positive regions in myopic eyes (but with no focal RNFL defects on photography/OCT and no asymmetry of the RNFL between the eyes on the temporal-superior-nasal-inferior-temporal map of the OCT)^33^, and with no VF defects within the Bjerrum area typical for glaucoma were classified as myopic group with SN RNFL defect. A typical representative case indicated for this study is shown in Fig. 4. The VF defects observed in myopic patients in the present study were either temporal or inferotemporal (IT) VF defects, which is commonly observed in myopic eyes. Myopic patients with SN RNFL defect with corresponding IT or temporal VF defects on either the Humphrey 24–2 test or the full-field 81 point screening test were classified as myopic group with both SN RNFL/IT VF defects. Eyes with only blind spot enlargement on the VF was excluded.

### Measurement of the optic disc tilt, torsion, and disc–fovea angle

To quantify the anatomical profiles of the optic disc, we calculated the disc tilt from the disc ovality ratio, disc torsion from the torsion degree, disc–fovea angle, and PPA area from the red-free RNFL photographs, and we calculated the tilt degree from the HRT images. The measurement parameters were described in our previous studies^8,34–38^.

We acquired the optic-disc color and red-free RNFL photographs using the standard settings on a non-mydriatic retinal camera (Kowa, Tokyo, Japan). When the images were not clear or taken with misalignment, the photographs were reacquired. The disc photographs and red-free images were evaluated independently in a masked fashion by two of the authors (HYP and MYC) in random order. The disc ovality ratio, disc torsion degree, and disc–fovea angle were measured on the photographs using National Institutes of Health image analysis software (ImageJ version 1.40; available at http://rsb.info.nih.gov/ij/index.html [in the public domain]; developed by Wayne Rasband, National Institutes of Health, Bethesda, MD, USA) (Supplemental Fig. 5).

We determined the disc ovality index using the tilt ratio (the ratio of the longest disc diameter to the shortest). The optic disc was classified as a tilted disc when its tilt ratio exceeded 1.30^39–41^. Disc torsion describes the deviation of the long disc axis from the vertical meridian, which is the vertical line perpendicular to a reference line connecting the fovea and the disc center. The angle between the vertical meridian and the long axis of the disc describes the degree of torsion. The optic disc was classified as having torsion when the degree of torsion was greater than 15°^25,42^. The disc–fovea angle was described as the angle between the optic disc and the fovea, measured by the angle between the reference line and a horizontal line through the disc center, as described previously.

The degrees of horizontal and vertical disc tilt were measured using the HRT III system^35,43^. Topographic images were obtained through dilated pupils. Acceptable intrascan standard deviation was set at <30 μm. We measured the extent of the temporal and vertical tilts on the HRT printouts using the ImageJ software, as described previously^19,23^. Horizontal disc tilt was defined as the angle between a horizontal line and a line drawn manually to connect the two points where the height profile and disc margin met. Vertical disc tilt was defined as the angle between a vertical line and the line connecting the two points where the height profile and disc margin met. Positive and negative values of the temporal disc tilt indicated temporal and nasal tilts, respectively. Similarly, positive and negative tilts on the vertical meridian indicate inferior and superior tilts, respectively.

### Imaging

Each patient underwent OCT imaging with a commercially available SD-OCT device (Spectralis, Heidelberg Engineering GmbH, Heidelberg, Germany). We used a radial scanning pattern comprising 15° of angularly equidistant high-resolution 24 B-scans. The scan pattern was centered on the clinical optic disc, and each B-scan was averaged for 35 single images with 768 A-scans per B-scan. For each technique, the operator checked for image quality, including SD-OCT proper B-scan positioning, in the image frame centering on the ONH and a quality score of >20. When necessary, the images were reacquired. Optic disc photographs and SD-OCT scans were acquired within 3 months of each other.

### Optic disc margin structures in spectral domain optical coherence tomography images

One trained observer (H.Y.P.) identified the optic disc margin and analyzed the ONH structures using OCT images, blinded to patient information. We modified this method from previously published studies^18,19,44^. An infrared image was extracted from the SD-OCT raw data. The best-quality photograph with clear focus at the disc margin was selected and rescaled to 1536 × 1536 pixels. We aligned the selected photograph to the corresponding infrared image to generate a registered photograph. For the exact alignment, we superimposed a 65% transparent photograph on the infrared image using Adobe Photoshop (Adobe Systems, San Jose, CA). Co-localization was checked by identifying the central retinal vessels and their bifurcations. Misalignments between the two images were corrected by scaling, rotating, and shifting the images. The demarcation of the optic disc margins in the registered photographs was performed using Adobe Photoshop. The observer also used the original stereophotograph to accurately identify the disc margin, defined as the innermost border of the reflective tissue that was internal to any pigmented tissue or the innermost termination of pigmented tissue when the reflective tissue was unclear. Two additional observers (S.J. and Y.J.) checked the optic discs and the location of the optic disc margin.

After identifying the optic disc margin on the photograph, we imported the raw SD-OCT data into software to visualize the 3-dimensional construction of the ONH and manually segment the SD-OCT structures onto the corresponding 2-dimentional registered photograph (Amira 5.2.2; Visage Imaging, Berlin, Germany). Using the registered photograph with the marked optic disc margin, one observer (H.Y.P.) identified the SD-OCT structures coinciding with the optic disc margin in all radial scans. The SD-OCT structures at the optic disc margin were categorized into BM overhang or border tissue overhang using the SD-OCT structure coinciding with the optic disc margin in the photograph. Data were presented for the 12 clock-hour positions in a right-eye format. The SD-OCT structure coinciding with the optic disc margin was classified into 3 categories first proposed by Burgoyne, C.F. and Chauhan, B.C.^14^: (1) BM opening (BMO), when the innermost edge of the BMO is co-localized with the clinical disc margin; (2) BM/border tissue, when the disc margin is co-localized with BM and border tissue, but the innermost edges of the two structures did not coincide; and (3) border tissue alone, when the disc margin co-localized with the border tissue alone. When border tissue alone was identified within the disc margin, this was defined as border tissue overhang.

### Evaluation of the reproducibility of clinical disc margin marking

To assess the reproducibility of disc margin delineation, a different observer (Y.J.) marked the clinical disc margin on the optic disc photographs for a subgroup of 20 eyes with the same method. The optic disc regions marked by two observers were saved for comparison. To quantify the extent of overlap, we used the Jaccard Index, which means we divided the size of the overlap between the two observers by the size of the union. A Jaccard Index ranges from 0 to 1; 1 indicates that two regions delineated by two observers are identical and lesser values means disagreement^45^.

### Statistical analyses

Comparison between eyes with and without VF defect was performed using independent *t*-test and comparison among three groups was performed using one-way ANOVA with multiple comparisons. The *chi*-square test was used where appropriate to compare frequencies. *P* values less than 0.05 were considered statistically significant. Statistical analyses were performed using the SPSS statistical package (SPSS, Chicago, IL, USA).

## Supplementary information

fig 5
