# Supplementary material for: Vertical disc tilt and features of the optic nerve head anatomy are related to visual field defect in myopic eyes
Source: Sci Rep. 2019 Mar 5;9:3485. doi: 10.1038/s41598-019-38960-6 (PMC6401376; doi:10.1038/s41598-019-38960-6)
Supplement: Supplementary file 1 — fig 5 [file 41598_2019_38960_MOESM1_ESM.pdf]

# Vertical disc tilt and features of the optic nerve head anatomy are related to visual field defect in myopic eyes

Hae-Young Lopilly Park, M.D., Ph.D, Yong Chan Kim, M.D.,

Younhea Jung, M.D., Ph.D., Chan Kee Park, M.D., Ph.D

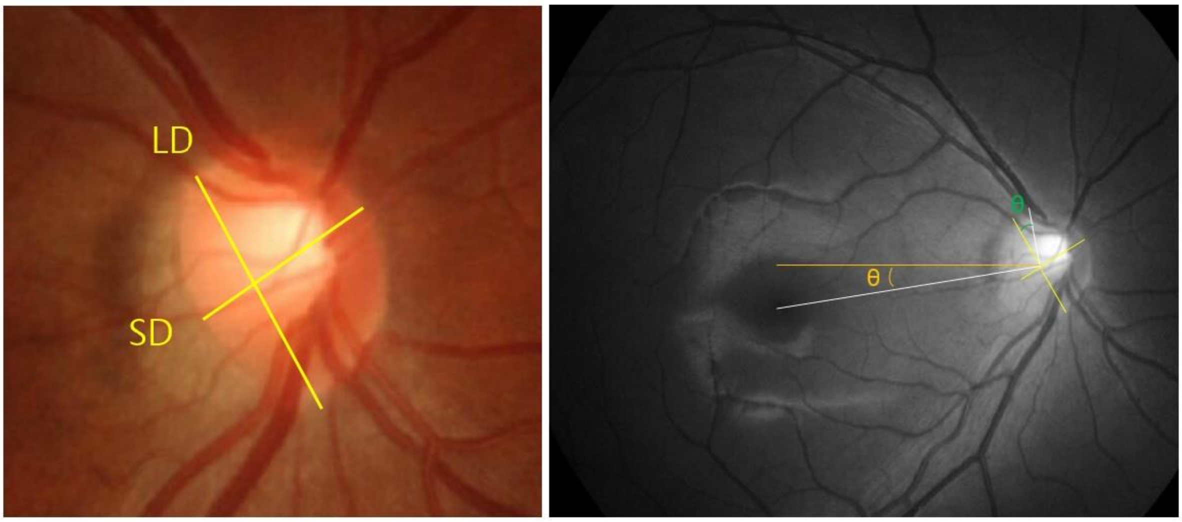


Supplemental Figure 5. Measurement of disc tilt, disc torsion, and disc-foveal angle. We determined the disc ovality index using the tilt ratio (the ratio of the longest disc diameter [LD] to the shortest diameter [SD]). Disc torsion describes the deviation of the long disc axis from the vertical meridian, which is the vertical line perpendicular to a reference line connecting the fovea and the disc center (white lines). The angle between the vertical meridian and the long axis of the disc describes the degree of torsion (green θ). The disc–fovea angle was described as the angle between the optic disc and the fovea, measured by the angle (orange θ) between the reference line and a horizontal line through the disc center (orange line).
